# Spermine and spermidine modulate T-cell function in older adults with and without cognitive decline ex vivo

**DOI:** 10.18632/aging.103527

**Published:** 2020-06-30

**Authors:** Maximilian Fischer, Johanna Ruhnau, Juliane Schulze, Daniela Obst, Agnes Flöel, Antje Vogelgesang

**Affiliations:** 1 Department of Neurology, University Medicine, Greifswald, Germany

**Keywords:** dementia, spermine, spermidine, T cell, cytokines

## Abstract

The global increase in neurodegenerative disorders is one of the most crucial public health issues. Oral polyamine intake was shown to improve memory performance which is thought to be mediated at least in part via increased autophagy induced in brain cells. In Alzheimer’s Disease, T-cells were identified as important mediators of disease pathology. Since autophagy is a central regulator of cell activation and cytokine production, we investigated the influence of polyamines on T-cell activation, autophagy, and the release of Th1/Th2 cytokines from blood samples of patients (n=22) with cognitive impairment or dementia in comparison to healthy controls (n=12) *ex vivo*. We found that spermine downregulated all investigated cytokines in a dose-dependent manner. Spermidine led to an upregulation of some cytokines for lower dosages, while high dosages downregulated all cytokines apart from upregulated IL-17A. Autophagy and T-cell activation increased in a dose-dependent manner by incubation with either polyamine. Although effects in patients were seen in lower concentrations, alterations were similar to controls.

We provide novel evidence that supplementation of polyamines alters the function of T-cells. Given their important role in dementia, these data indicate a possible mechanism by which polyamines would help to prevent structural and cognitive decline in aging.

## INTRODUCTION

The most common form of dementia is the Alzheimer's Disease (AD) type. Clinically, it progresses from subjective cognitive decline (SCD) to mild cognitive impairment (MCI) and finally to the dementia stage. Due to the increasing life expectancy of the world's population, current studies predict a dramatic increase in the number of people affected by cognitive diseases by the year 2050 [1]. This burden is aggravated by very limited treatment options for AD dementia which includes two medical approaches only: cholinesterase inhibitors and NMDA-receptor-antagonists [2]. Therefore, it is important to explore prevention and early treatment strategies for this disorder.

Polyamines are a group of organic compounds characterized by a terminal amino group and an alternating number of secondary amino groups. The most important representatives of this class include spermidine, spermine, cadaverine, and putrescine. The natural polyamines spermidine and spermine are present in all eukaryotic cells supporting essential functions in cell proliferation and differentiation as well as immune regulation [3]. Effects of polyamines on cognition were reported from several animal models and from humans: i) Supplementation with polyamines improved memory functions of aged flies [4]; ii) Higher polyamine levels in the hippocampus were shown to correlate with better memory retrieval and formation [5], and changes in polyamine levels in memory-associated brain structures are suggested to contribute to age-related impairments in learning and memory in rats [6]; iii) Encouraging results have been reported from a proof of concept trial, showing that 3-months supplementation with a plant extract mainly containing spermidine and spermine (with 1.2 mg spermidine and 0.6 mg spermine per 1 g extract) as active substances was not only safe [3], but also improved memory functions of older adults with SCD [7]. Based on these data, an ongoing trial investigates the effects of a 12-months polyamine supplementation in patients with SCD (SmartAge) [8].

However, our knowledge about possible mechanisms of action of polyamines in cognitive diseases is very limited so far. One possible mode of action might be their known induction of autophagy [9, 10]. Increased autophagy was shown to be associated with improvement of cognitive decline in vascular dementia [11]. Autophagy was also shown to regulate microglia polarization in neurodegeneration [12]. The positive effects are reviewed by Madeo et al. [10].

In addition to these direct actions on brain cells, polyamines might also influence the function of immune cells. The importance of immune cells for cognition is well documented: CD4+ T-cells regulate the brain immune response through the secretion of cytokines and are involved in effective clearance of Aß-plaques. Through the secretion of inflammatory cytokines, like tumor necrosis factor-α (TNF-α) or Interferon-γ (INF-γ), they can induce inflammation and neurodegeneration, but also protect neurons through the secretion of anti-inflammatory cytokines like Interleukin-10 (IL-10) [13].

But how would polyamines influence T cell function? Modulation of autophagy might be the decisive mechanism, since T-cells display decreased autophagic activity in aging organisms [14]. To date, the available data with regard to immunoregulatory influence of polyamines on T-cell differentiation are controversial [15–18]. Differences between previous reports might be explained by i) different cell culture conditions, ii) the application of different readout methods for proliferation and activation of cells, and iii) differences in the compartments from which T-cells were isolated. With regard to activation marker and cytokine release, spermidine was reported to decrease cell adhesion by selectively decreasing lymphocyte function-associated antigen 1 (LFA-1), which is involved in immune cell activation and inflammation [19]. However, other authors reported spermine to inhibit the synthesis of proinflammatory cytokines like TNF, IL-1, IL-6, macrophage inflammatory protein-α (MIP-1α), and MIP-1β in lipopolysaccharide treated human peripheral blood mononuclear cells (PBMC) [20]. In addition, previous data have suggested a positive effect of spermidine in memory T-cell response formation [21, 22].

Taken together, the exact mechanisms by which spermidine may ameliorate cognitive decline or may promote brain health are still incompletely understood. Polyamines might exert their positive effects on cognition through the direct induction of autophagy in brain cells, but also indirectly through modulation of immune cell function like CD4+ T-cells which then invade the brain and modulate brain cell function through direct cell to cell interaction or indirect via, for example, cytokine secretion. While it is known that autophagy modulates T-cell activation and cytokine production, both T-cell activation and cytokine milieu can also regulate autophagy [14, 23] and it is unclear from which direction the detrimental interaction starts in AD pathology. We therefore analyzed the influence of spermine and spermidine on T-cell autophagy, T-cell activation – as determined by surface CD25 and CD69 – and on release of cytokines by PBMC in blood samples from patients with cognitive decline (CD-patients: SCD, MCI and early dementia) and in healthy age-matched controls. We considered two possible avenues: i) low concentrations of polyamines in blood, as reached by oral supplementation, might be sufficient to induce alterations. This option was covered by the low concentrations applied in our cell culture (5 and 10μM); ii) higher concentrations of polyamines, as reached in the gut where resident T-cells get altered in their function and invade the brain, might be decisive. The interaction of gut and brain through modulation of immune function [24] and trafficking [25] is well-documented as part of the gut-brain-axis in several neurological diseases [26]. To cover this option, higher concentrations of spermine and spermidine (100 to 2000μM) were included.

## RESULTS

### Spermine and spermidine alter cytokine release

Cytokines were quantified after 48 hours of ex vivo stimulation with either spermine or spermidine.

### Spermine decreases all cytokines

**Figure 1:**
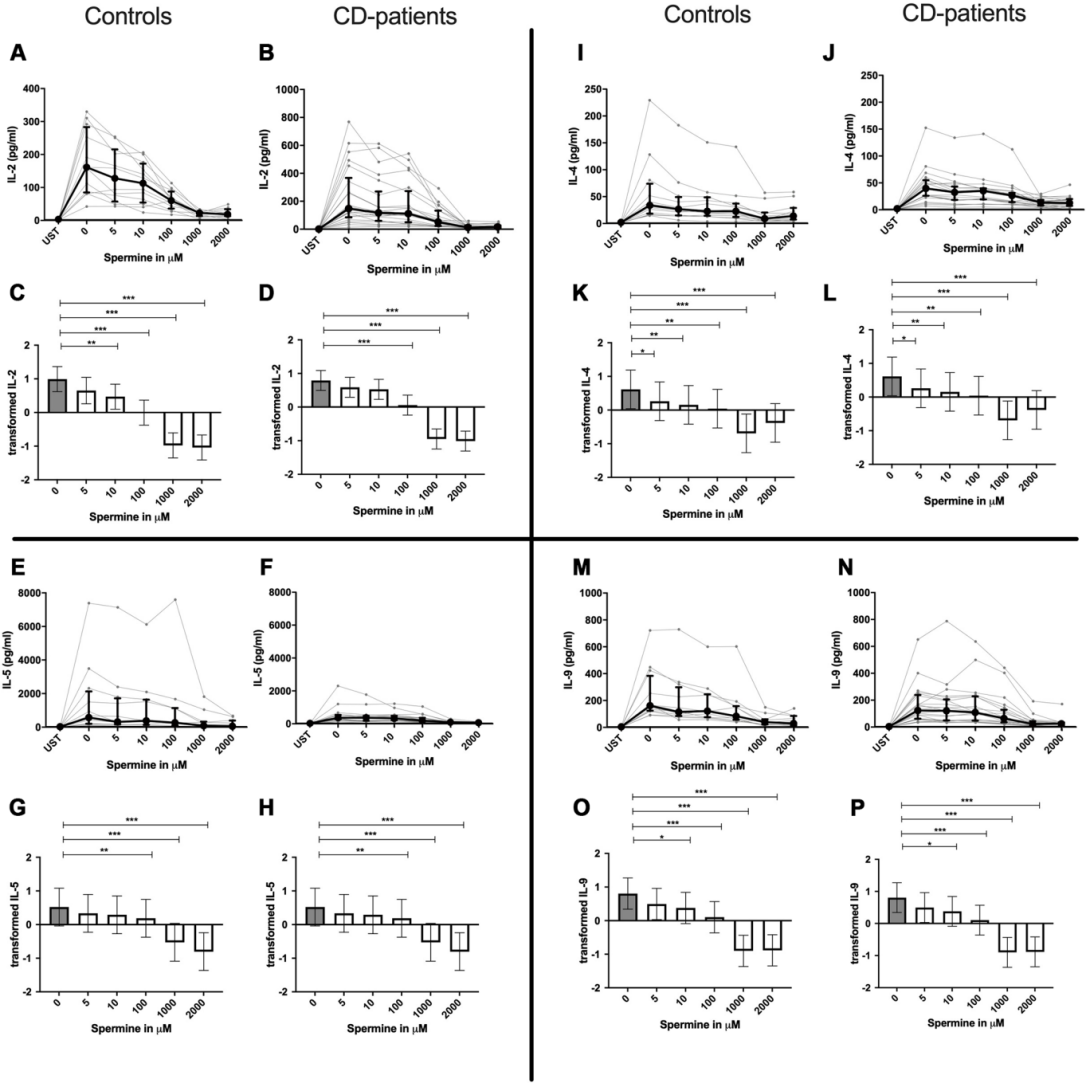
**Cytokine data (IL2, IL-4, IL-5, IL-9) of pre-stimulated T-cell (24 hours) treated with and without Spermine (in pg/ml) for 48 hours in cognitive decline patients and controls.** Cytokines – IL2, IL-4, IL-5, IL-9– in pg/ml – were measured by Legend-plex (Biolegend) in culture supernatants after 48 hours of Spermine treatment. T-cells were pre-stimulated by CD3/CD28 antibodies for 24 hours before polyamine usage. Spermine was added in the following concentrations: 5μM, 10μM, 100μM, 1000μM, 2000μM. Unstimulated cells (UST) were measured as control. The data sets were not Gaussian-distributed and therefore transformed by R studio orderNorm using the bestNormalize package and analyzed. Raw data (individual data, median + interquantile ranges) (**A**, **B**, **I**, **J**, **E**, **F**, **M**, **N**) and transformed data are given (mean with lower and upper confidence limits, **C**, **D**, **K**, **L**, **G**, **H**, **O**, **P**). n_control_ = 12; n_CD-patient_ = 20 * p<0.05; ** p<0.01; *** p<0.001.

**Figure 2:**
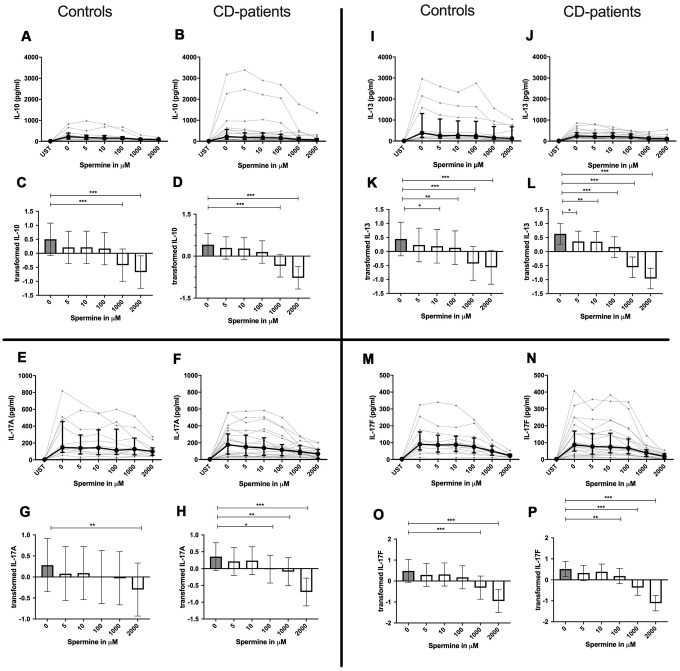
**Cytokine data (IL-10, IL-13, IL17A, IL17-F) of pre-stimulated T-cell (24 hours) treated with and without Spermine (in pg/ml) for 48 hours in cognitive decline patients and controls.** Cytokines – IL-10, IL-13, IL-17A, IL-17F– in pg/ml – were measured by Legend-plex (Biolegend) in culture supernatants after 48 hours of Spermine treatment. T-cells were pre-stimulated by CD3/CD28 antibodies for 24 hours before polyamine usage. Spermine was added in the following concentrations: 5μM, 10μM, 100μM, 1000μM, 2000μM. Unstimulated cells (UST) were measured as control. The data sets were not Gaussian-distributed and therefore transformed by R studio orderNorm using the bestNormalize package and analyzed. Raw data (individual data, median + interquantile ranges) (**A**, **B**, **I**, **J**; **E**, **F**, **M**, **N**) and transformed data are given (mean with lower and upper confidence limits, **C**, **D**, **K**, **L**, **G**, **H**, **O**, **P**). n_control_ = 12; n_CD-patient_ = 20 * p<0.05; ** p<0.01; *** p<0.001.

**Figure 3:**
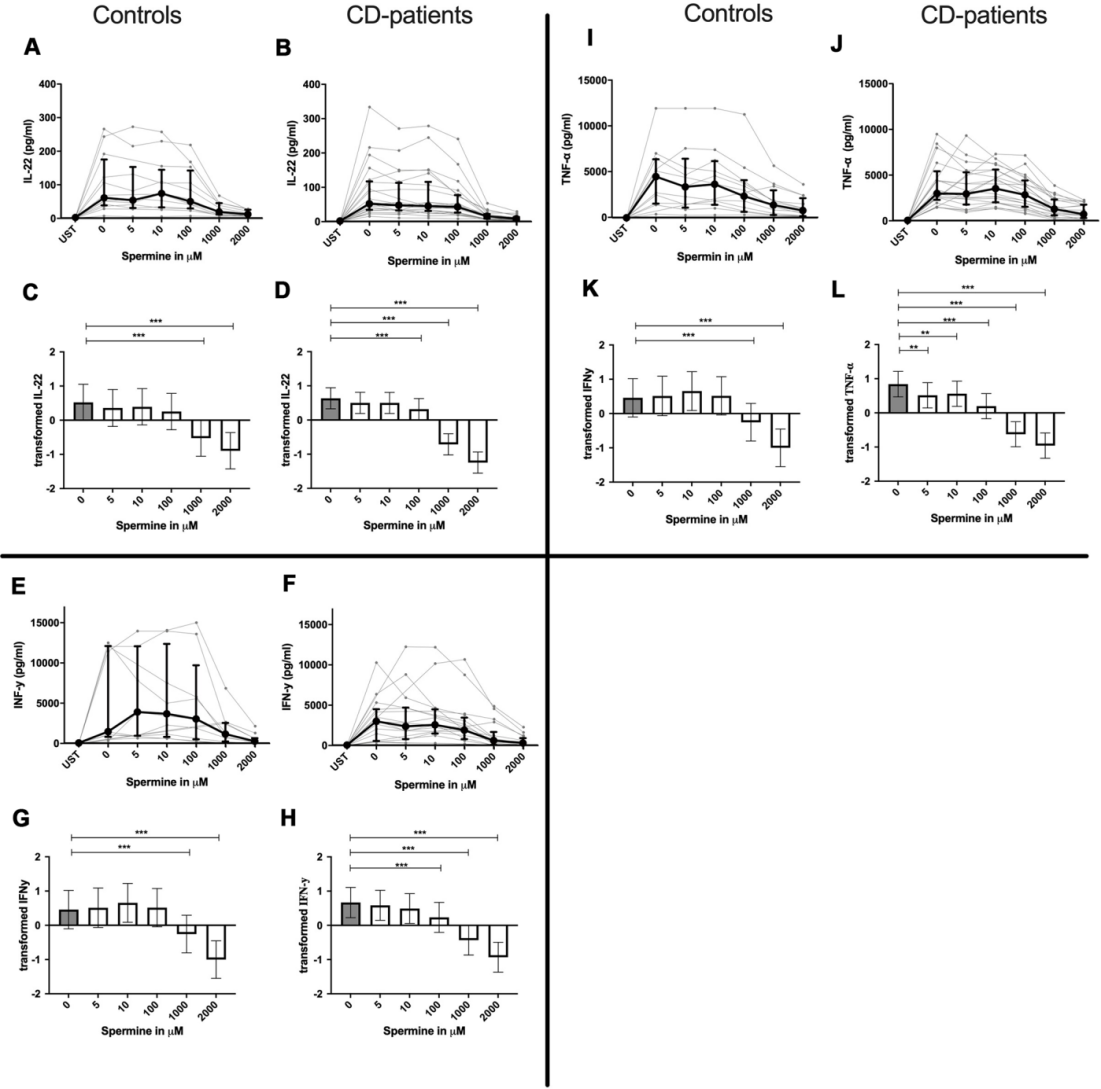
**Cytokine data (IL-22, INF-γ, TNF-α) of pre-stimulated T-cell (24 hours) treated with and without Spermine (in pg/ml) for 48 hours in cognitive decline patients and controls.** Cytokines – IL22, INF-γ, TNF-α – were measured by Legend-plex (Biolegend) in culture supernatants after 48 hours of Spermine treatment. T-cells were pre-stimulated by CD3/CD28 antibodies for 24 hours before polyamine usage. Spermine was used in the following concentrations: 5μM, 10μM, 100μM, 1000μM, 2000μM. Unstimulated cells (UST) were measured as control. The data sets were not Gaussian-distributed and therefore transformed by R studio orderNorm using the bestNormalize package and analyzed. Raw data (individual data, median + interquantile ranges) (**A**, **B**, **I**, **J**, **E**, **F**) and transformed data are given(mean with lower and upper confidence limits, **C**, **D**, **K**, **L**, **G**, **H**). n_control_ = 12; n_CD-patient_ = 20 * p<0.05; ** p<0.01; *** p<0.001.

Addition of spermine caused downregulation of all cytokines. The effects are reported from the lowest to the highest dosage:

IL-4, IL-13 and TNF-α production were reduced in CD-patients samples dose-dependently from 5μM of spermine upwards, in control samples reduction occurred not before 10μM for IL-13 and TNF-α. IL-2 and IL-9 were reduced from 10μM of spermine upwards in control samples. However, the reduction was not evident in CD-patients before 100μM and upwards. IL-17A, IL-17F, IL-22 and INF-γ were downregulated in CD-patients starting from 100μM of spermine. The reduction was not apparent in controls for IL-17F, IL-22 and INF-γ until 1000μM or 2000μM, for IL-17A even first at 2000μM. IL-5 was downregulated in both groups from 100μM to 2000μM. IL-10 was only downregulated at 1000μM and 2000μM of spermine in both groups. Cytokines are displayed in alphabetical order, Figure 1: IL-2 to IL-9, Figure 2: IL-10 to IL-17F, Figure 3: IL-22 to IFN-γ, Supplementary Table 1.

### Spermidine increases or decreases cytokines depending on dosage

In contrast to spermine, addition of spermidine caused a dose-dependent decrease, increase, or a combination of the two. For IL-4, IL-10 and IL-22 only downregulation could be observed at different onset concentrations of spermidine and with a higher sensitivity in CD-patients for IL-22. In contrast IL-17A was consistently and increasingly upregulated in both groups from 10μM upwards.

**Figure 4:**
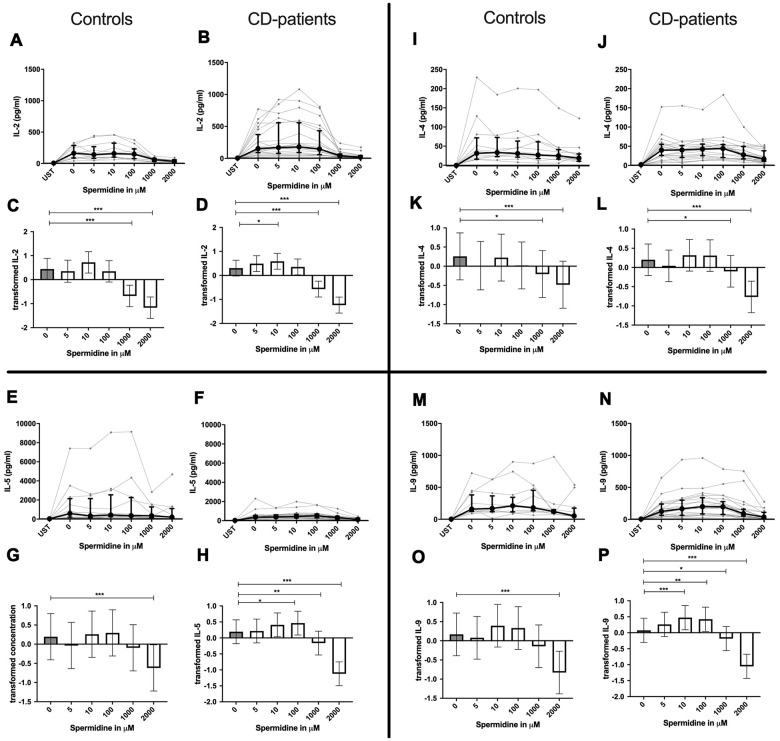
**Cytokine data (IL-2, IL-4, IL-5, IL-9) of pre-stimulated T-cell (24 hours) treated with and without Spermidine (in pg/ml) for 48 hours in cognitive decline patients and controls.** Cytokines – IL-2, IL-4, IL-5, IL-9 – were measured by Legend-plex (Biolegend) in culture supernatants after 48 hours of Spermidine treatment. T-cells were pre-stimulated by CD3/CD28 antibodies for 24 hours before polyamine usage. Spermidine was used in the following concentrations: 5μM, 10μM, 100μM, 1000μM, 2000μM. Unstimulated cells (UST) were measured as control. The data sets were not Gaussian-distributed and therefore transformed by R studio orderNorm using the bestNormalize package and analyzed. Raw data (individual data, median + interquantile ranges) (**A**, **B**, **I**, **J**; **E**, **F**, **M**, **N**) and transformed data are given (mean with lower and upper confidence limits, **C**, **D**, **K**, **L**, **G**, **H**, **O**, **P**). n_control_ = 12; n_CD-patient_ = 20 * p<0.05; ** p<0.01; *** p<0.001.

**Figure 5:**
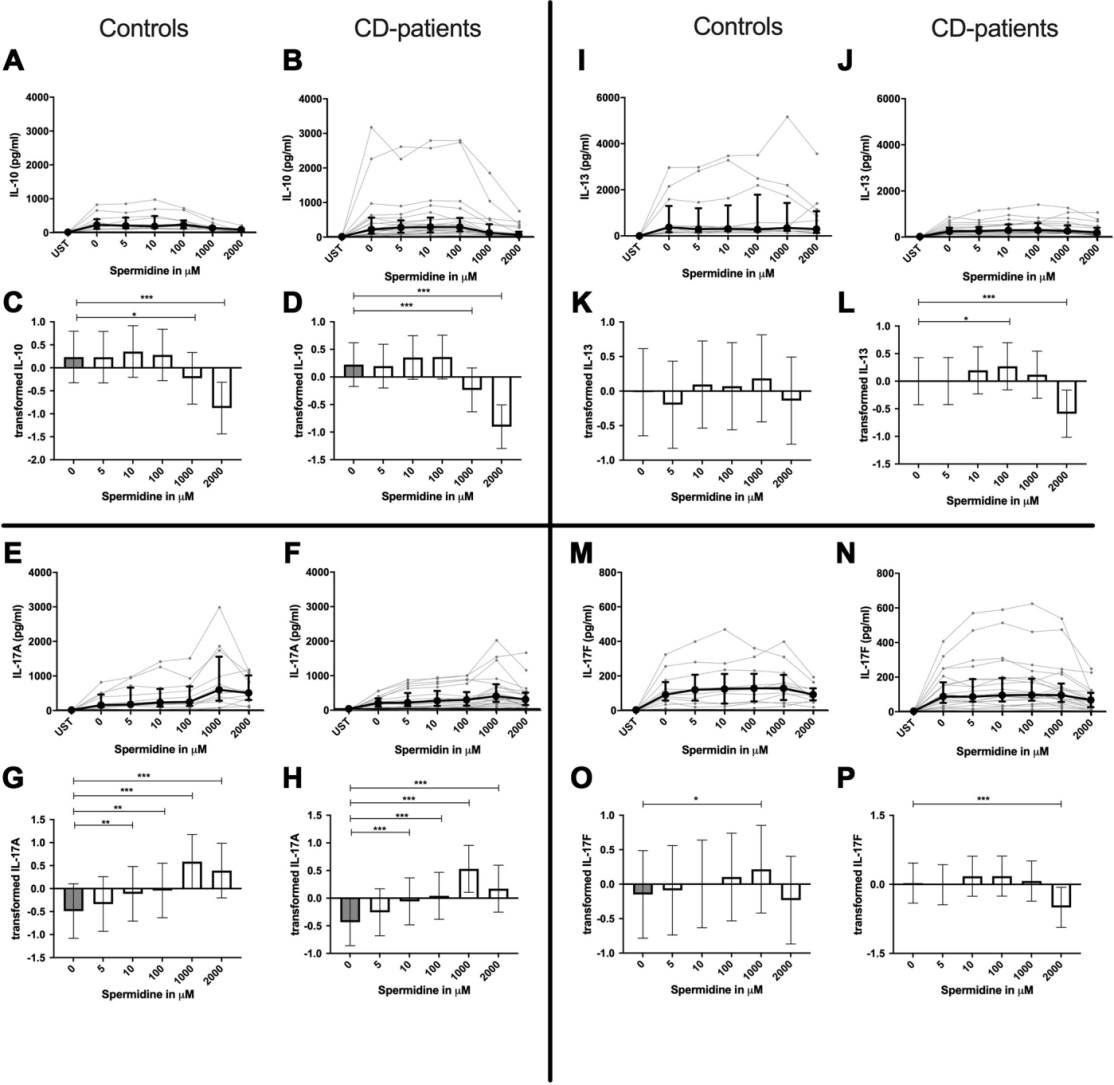
**Cytokine data (IL-10, IL-13, IL-17A, IL-17F) of pre-stimulated T-cell (24 hours) treated with and without Spermidine (in pg/ml) for 48 hours in cognitive decline patients and controls.** Cytokines –IL-10, IL-13, IL-17A, IL-17F– were measured by Legend-plex (Biolegend) in culture supernatants after 48 hours of Spermidine treatment. T-cells were pre-stimulated by CD3/CD28 antibodies for 24 hours before polyamine usage. Spermidine was used in the following concentrations: 5μM, 10μM, 100μM, 1000μM, 2000μM. Unstimulated cells (UST) were measured as control. The data sets were not Gaussian-distributed and therefore transformed by R studio orderNorm using the bestNormalize package and analyzed. Raw data (individual data, median + interquantile ranges) (**A**, **B**, **I**, **J**; **E**, **F**, **M**, **N**) and transformed data are given (mean with lower and upper confidence limits, **C**, **D**, **K**, **L**, **G**, **H**, **O**, **P**). n_control_ = 12; n_CD-patient_ = 20 * p<0.05; ** p<0.01; *** p<0.001.

**Figure 6:**
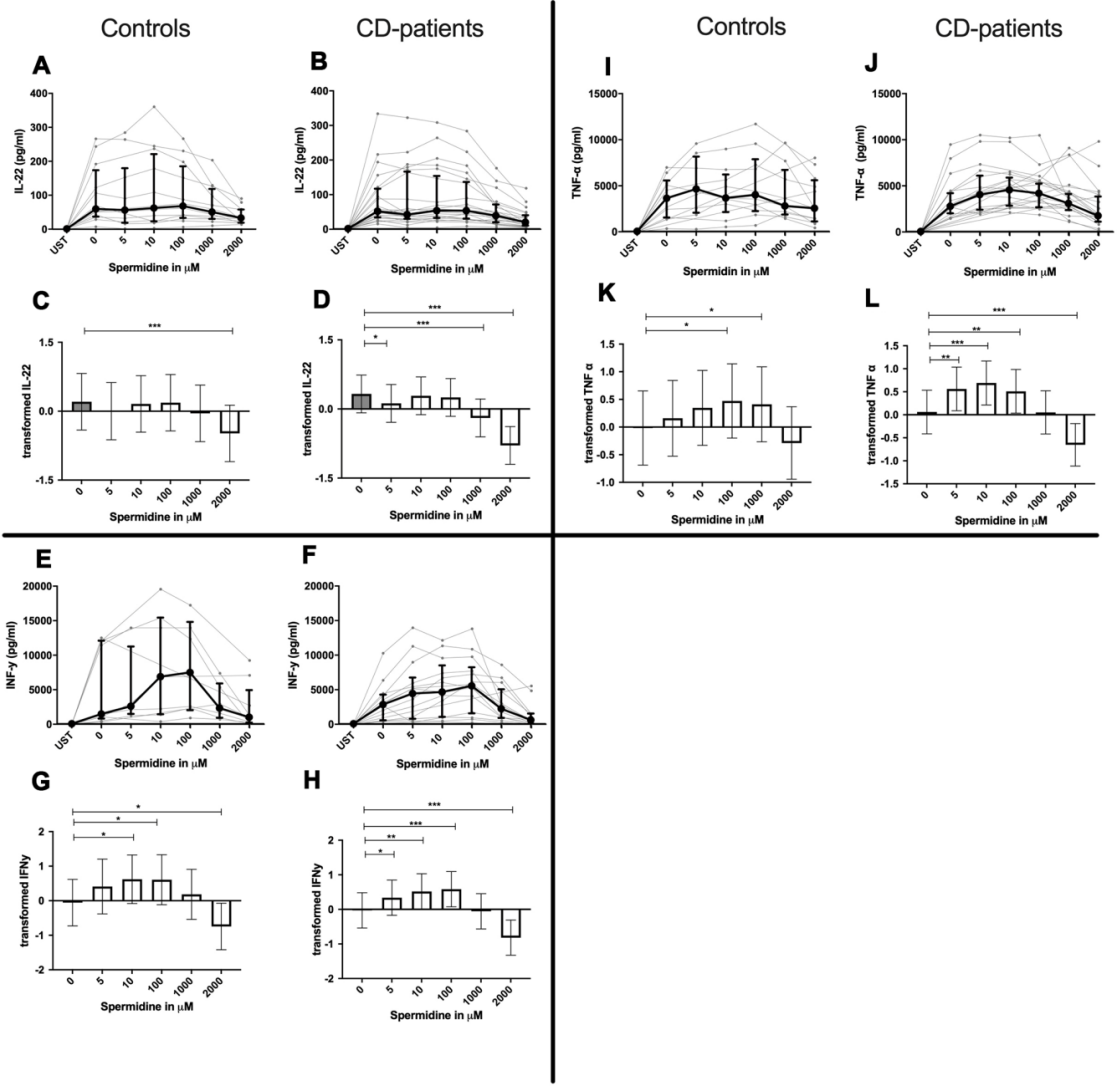
**Cytokine data (IL-22, INF-γ, TNF-α) of pre-stimulated T-cell (24 hours) treated with and without Spermidine (in pg/ml) for 48 hours in cognitive decline patients and controls.** Cytokines – IL-22, INF-γ, TNF-α – were measured by Legend-plex (Biolegend) in culture supernatants after 48 hours of Spermidine treatment. T-cells were pre-stimulated by CD3/CD28 antibodies for 24 hours before polyamine usage. Spermidine was used in the following concentrations: 5μM, 10μM, 100μM, 1000μM, 2000μM. Unstimulated cells (UST) were measured as control. The data sets were not Gaussian-distributed and therefore transformed by R studio orderNorm using the bestNormalize package and analyzed. Raw data (individual data, median + interquantile ranges) (**A**, **B**, **I**, **J**, **E**, **F**) and transformed data are given (mean with lower and upper confidence limits, **C**, **D**, **K**, **L**, **G**, **H**). n_control_ = 12; n_CD-patient_ = 20 * p<0.05; ** p<0.01; *** p<0.001.

IL-2, IL-5, IL-9, IL-13, IL-17F, INF-γ and TNF-α in contrast showed both up- and downregulation - depending on the dosage. Lower dosages up to 100μM increased cytokine, and higher dosages had either no effect or led to cytokine decrease. In both extremes, CD-patient’s cells seemed to be more sensitive to spermidine. Cytokines are displayed in alphabetical order, Figure 4: IL-2 to IL-9, Figure 5: IL-10 to IL-17F, Figure 6: IL-22 to IFN-γ, Supplementary Table 1.

Summary: Spermine downregulated all cytokines in a dose-dependent manner. Spermidine in contrast led to upregulation of IL-2, IL-5, IL-9, IL-17A, INF-γ and TNF-α for lower dosages, while high dosages only upregulated IL-17A. All other cytokines were downregulated.

### Spermine and spermidine increase CD4+ and CD8+ T-cell activation as well as autophagy

### Spermine influence on CD4+ and CD8+ T-cells

**Figure 7:**
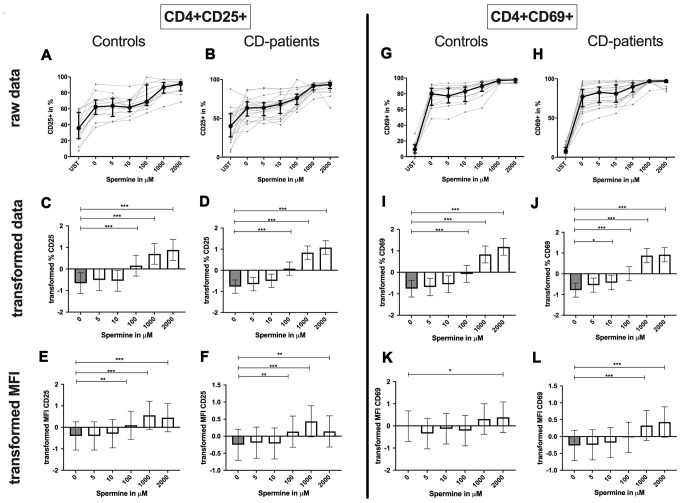
**Activation marker CD25, CD69 of CD4+ T helper-cells treated with Spermine for cognitive decline patients and controls.** T-cells were isolated from controls (**A**, **C**, **E**, **G**, **I**, **K**) and patients with cognitive decline (**B**, **D**, **F**, **H**, **J**, **L**). CD3/CD28 stimulated T-cells were incubated for 24 hours with 5, 10, 100, 1000, 2000 μM Spermine. Percentage (%) (**A**–**D**, **G**–**J**) and the expression (measured by median fluorescence intensity; MFI) (**E**, **F**, **K**, **L**) of activation marker – CD25 (**A**–**F**); CD69 (**G**–**L**). Markers were analysed on CD4+ T-cells. The data sets were not Gaussian-distributed and therefore transformed by R studio orderNorm using the bestNormalize package and analyzed. Raw data (individual data, median + interquantile ranges) and transformed data (mean with lower and upper confidence limits) are depicted for %. n_control_ = 12; n_CD-patient_ = 20; * p<0.05; ** p<0.01; *** p<0.001.

**Figure 8:**
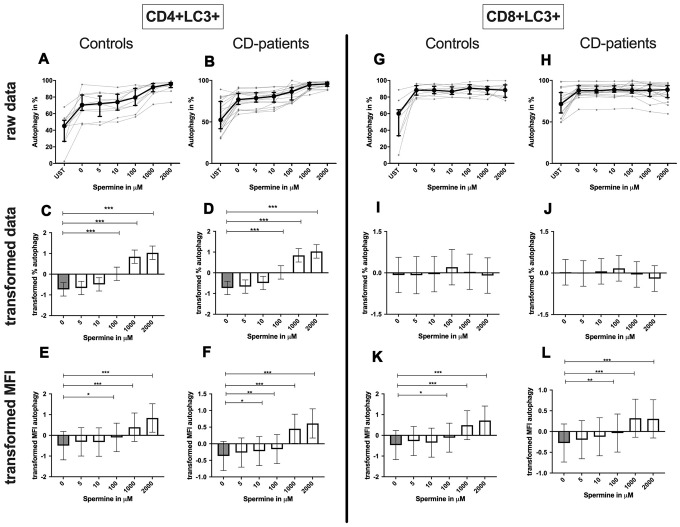
**Autophagy of CD4+ T helper-cells and cytotoxic CD8+ T-cells treated with Spermine for cognitive decline patients and controls.** T-cells were isolated from controls (**A**, **C**, **E**, **G**, **I**, **K**) and patients with cognitive decline (**B**, **D**, **F**, **H**, **J**, **L**). CD3/CD28 stimulated T-cells were incubated for 24 hours with 5, 10, 100, 1000, 2000 μM Spermine. Percentage (%) (**A**–**D**, **G**–**J**) and the expression (measured by median fluorescence intensity; MFI) (**E**, **F**, **K**, **L**) of autophagy was measured by LC3. Autophagy was analysed on CD4+ T-cells (**A**–**F**) and CD8+ T-cells (**G**–**L**). The data sets were not Gaussian-distributed and therefore transformed by R studio orderNorm using the bestNormalize package and analyzed. Raw data (individual data, median + interquantile ranges) and transformed data (mean with lower and upper confidence limits) are depicted for %. n_control_ = 12; n_CD-patient_ = 20; * p<0.05; ** p<0.01; *** p<0.001.

**CD4+ T-cells:** Spermine-treated samples showed a dose-dependent increase in the percentage of CD4+ CD25+ and CD4+ CD69+ T-cells for 100μM of spermine upwards in CD-patients and controls. The percentage of CD4+ CD69+ T-cells was significantly increased from 10μM compared to no spermine condition in CD-patient samples only. (Figure 7: 7A–7D for CD4+CD25+; Figure 7G–7J for CD4+CD69+) While higher concentrations of spermine induced a higher amount of CD25 per cell (as measured by MFI) in CD-patients and controls from 100μM upwards, CD69 increased already at lower concentrations in CD-patients than in controls (Figure 7: 7E, 7F for CD4+CD25; Figure 7K, 7L for CD4+CD69+; Supplementary Figure 1A–1D). Spermine induced an increased percentage of autophagic CD4+ T-cells derived from CD-patients and controls from 100μM upwards (Figure 8A–8D). The amount of the autophagy marker LC3 per cell increased in all concentrations from 10μM upwards in CD patients. Autophagy increase per cell in control samples could not be observed before 100μM and upwards. (Figure 8E, 8F; Supplementary Figure 1E, 1F)

**Figure 9:**
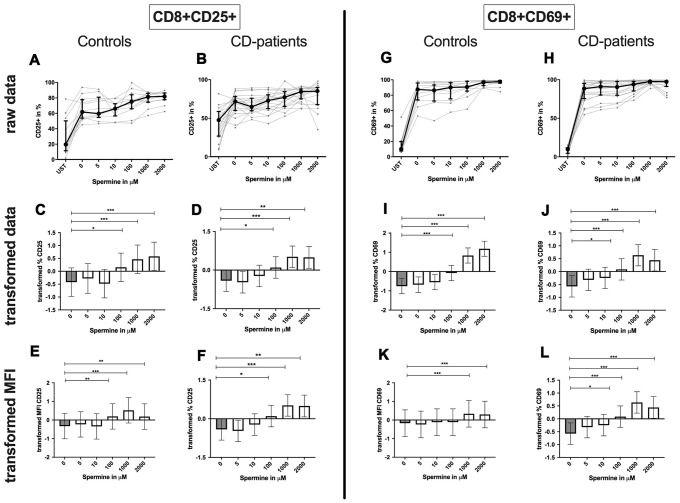
**Activation marker CD25, CD69 of cytotoxic CD8+ T-cells treated with Spermine for cognitive decline patients and controls.** T-cells were isolated from controls (**A**, **C**, **E**, **G**, **I**, **K**) and patients with cognitive decline (**B**, **D**, **F**, **H**, **J**, **L**). CD3/CD28 stimulated T-cells were incubated for 24 hours with 5, 10, 100, 1000, 2000 μM Spermine. Percentage (%) (**A**–**D**, **G**–**J**) and the expression (measured by median fluorescence intensity; MFI) (**E**, **F**, **K**, **L**) of activation marker – CD25 (**A**–**F**); CD69 (**G**–**L**). Markers were analysed on CD8+ T-cells. The data sets were not Gaussian-distributed and therefore transformed by R studio orderNorm using the bestNormalize package and analyzed. Raw data (individual data, median + interquantile ranges) and transformed data (mean with lower and upper confidence limits) are depicted for %. n_control_ = 12; n_CD-patient_ = 20; * p<0.05; ** p<0.01; *** p<0.001.

**CD8+ T-cells:** A dose-dependent increase of CD8+ CD25+ or CD8+ CD69+ T-cell percentage could be observed from 100μM or 10μM respectively upwards in CD-patients and controls (Figure 9: 9A–9D for CD8+CD25+, Figure 9G–9J for CD8+CD69+ expression). The amount of CD25+ was enhanced in CD-patients and control samples from 100μM while the amount of CD69 per cell was only regulated for controls (Figure 9: 9E, 9F for CD4+CD25; Figure 9K, 9L for CD4+CD69+; Supplementary Figure 1G–1J).

Although the percentage of autophagic CD8+ T-cells was not altered by spermine, the amount of LC3 per cell increased with higher spermine concentrations in CD-patients and controls (Figure 8G–8L, Supplementary Figure 1K, 1L).

Summary: Autophagy, percentage of CD25 and CD69 expressing CD4+ or CD8+ T-cells as well as the amount of these activation markers per cell, increased in a dose-dependent manner by incubation with spermine in CD-patients and control samples.

### Spermidines influence on CD4+ and CD8+ T-cells

**Figure 10:**
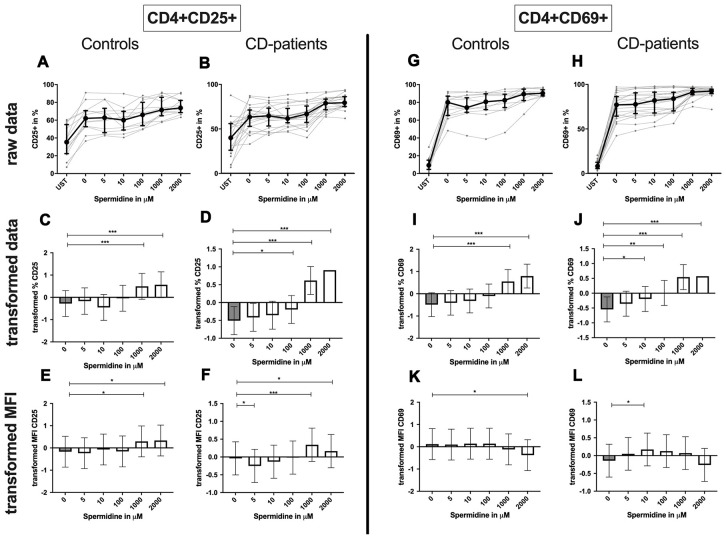
**Activation marker CD25, CD69 of CD4+ T helper-cells treated with Spermidine for cognitive decline patients and controls.** T-cells were isolated from controls (**A**, **C**, **E**, **G**, **I**, **K**) and patients with cognitive decline (**B**, **D**, **F**, **H**, **J**, **L**). CD3/CD28 stimulated T-cells were incubated for 24 hours with 5, 10, 100, 1000, 2000 μM Spermidine. Percentage (%) (**A**–**D**, **G**–**J**) and the expression (measured by median fluorescence intensity; MFI) (**E**, **F**, **K**, **L**) of activation marker – CD25 (**A**–**F**); CD69 (**G**–**L**). Markers were analysed on CD4+ T-cells. The data sets were not Gaussian-distributed and therefore transformed by R studio orderNorm using the bestNormalize package and analyzed. Raw data (individual data, median + interquantile ranges) and transformed data (mean with lower and upper confidence limits) are depicted for %. n_control_ = 12; n_CD-patient_ = 20; * p<0.05; ** p<0.01; *** p<0.001.

**CD4+ T-cells:** Spermidine-induced increase of CD4+ CD25+ and CD4+ CD69+ T-cell percentage in CD-patients at lower concentrations than in controls (Figure 10: 10A–10D for CD4+ CD25+, Figure 10G–10J for CD4+ CD69+ expression).

The amount of CD25 per T-cells was significantly increased in CD-patients and controls for 1000μM and 2000μM. In contrast, only for 5μM the amount of CD25 was reduced in CD-patients. CD69 on the T-cell surface was only reduced in controls treated with 2000μM Spermidine while 10 μM increased the amount of CD69 in CD-patients (Figure 10: 10E, 10F for CD4+CD25; Figure 10K, 10L for CD4+CD69+; Supplementary Figure 2A–2D).

**Figure 11:**
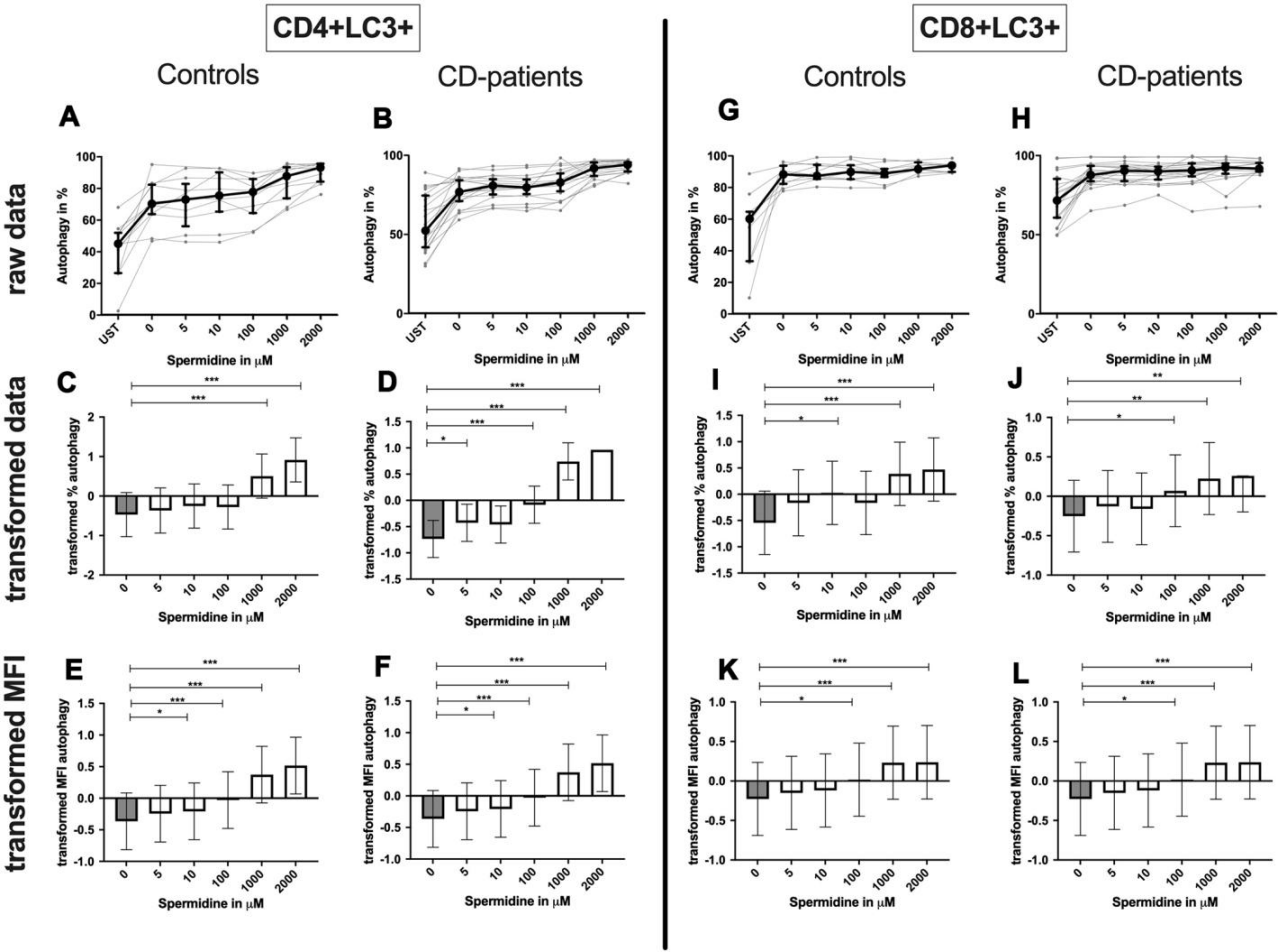
**Autophagy of CD4+ T helper-cells and cytotoxic CD8+ T-cells treated with Spermidine for cognitive decline patients and controls.** T-cells were isolated from controls (**A**, **C**, **E**, **G**, **I**, **K**) and patients with cognitive decline (**B**, **D**, **F**, **H**, **J**, **L**). CD3/CD28 stimulated T-cells were incubated for 24 hours with 5, 10, 100, 1000, 2000 μM Spermidine. Percentage (%) (**A**–**D**, **G**–**J**) and the expression (measured by median fluorescence intensity; MFI) (**E**, **F**, **K**, **L**) of autophagy was measured by LC3. Autophagy was analysed on CD4+ T-cells (**A**–**F**) and CD8+ T-cells (**G**–**L**). The data sets were not Gaussian-distributed and therefore transformed by R studio orderNorm using the bestNormalize package and analyzed. Raw data (individual data, median + interquantile ranges) and transformed data (mean with lower and upper confidence limits) are depicted for %. n_control_ = 12; n_CD-patient_ = 20; * p<0.05; ** p<0.01; *** p<0.001.

Spermidine increased the percentage of autophagic CD4+ T-cells more drastically in samples derived from CD-patients (starting at 5μM); controls only showed an induction for 1000μM and 2000μM (Figure 11A–11D). The amount of LC3 per cell was enhanced in both groups from 10μM (Figure 11E, 11F; Supplementary Figure 1E, 1F).

**Figure 12:**
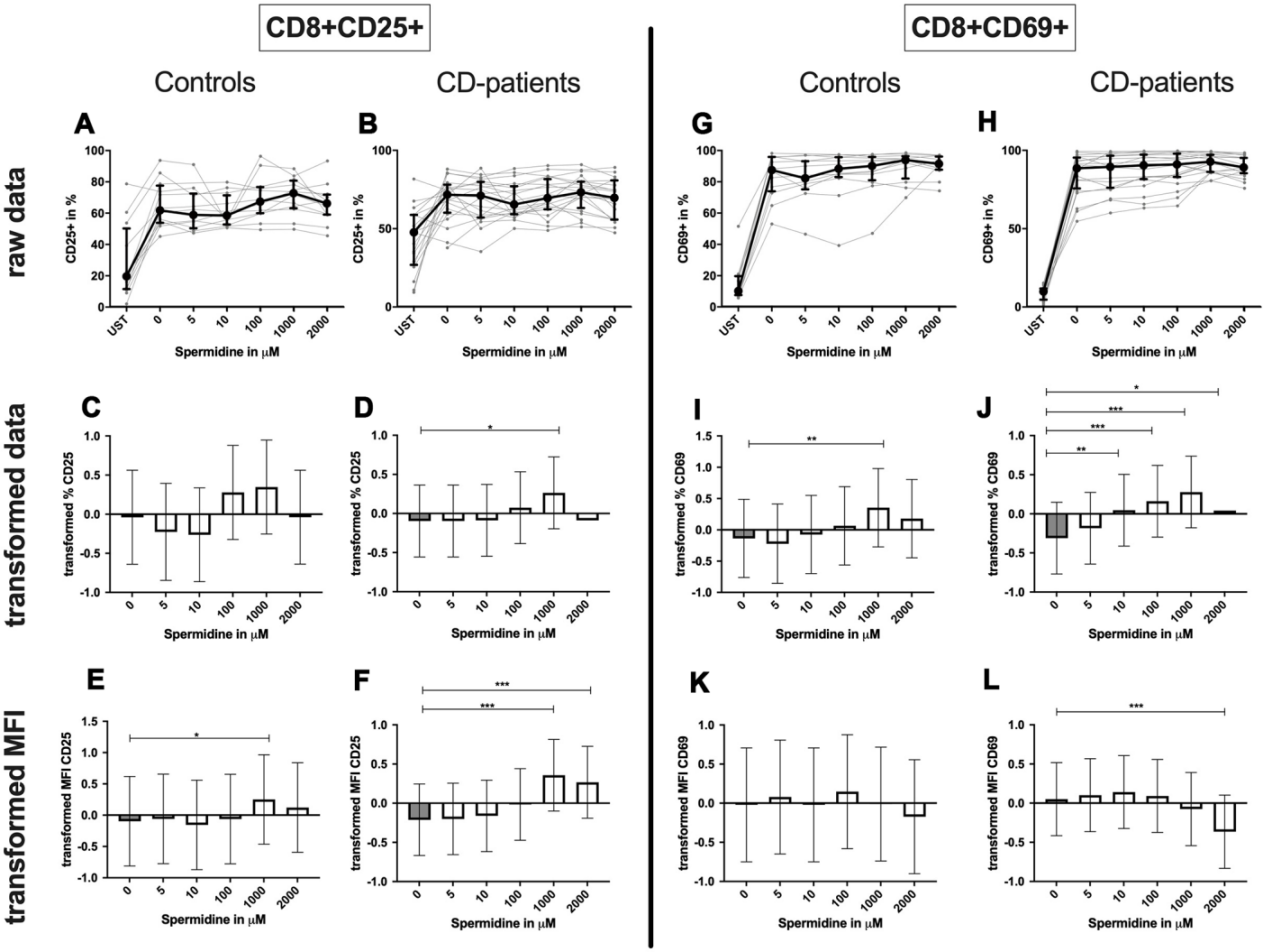
**Activation marker CD25, CD69 of cytotoxic CD8+ T-cells treated with Spermidine for cognitive decline patients and controls.** T-cells were isolated from controls (**A**, **C**, **E**, **G**, **I**, **K**) and patients with cognitive decline (**B**, **D**, **F**, **H**, **J**, **L**). CD3/CD28 stimulated T-cells were incubated for 24 hours with 5, 10, 100, 1000, 2000 μM Spermidine. Percentage (%) (**A**–**D**, **G**–**J**) and the expression (measured by median fluorescence intensity; MFI) (**E**, **F**, **K**, **L**) of activation marker – CD25 (**A**–**F**); CD69 (**G**–**L**). Markers were analysed on CD8+ T-cells. The data sets were not Gaussian-distributed and therefore transformed by R studio orderNorm using the bestNormalize package and analyzed. Raw data (individual data, median + interquantile ranges) and transformed data (mean with lower and upper confidence limits) are depicted for %. n_control_ = 12; n_CD-patient_ = 20; * p<0.05; ** p<0.01; *** p<0.001.

**CD8+ T-cell:** Only 1000μM of spermidine increased the percentage of activated CD25+ CD8+ T-cell in CD-patients but not in controls (Figure 12: 12A–12D for CD8+CD25+, Figure 12G–12J for CD8+CD69+ expression). The surface CD25 amount per cell was enhanced for 1000μM and 2000μM in CD-patients, but for 1000μM in controls. Spermidine also altered the percentage of CD69+ CD8+ T-cells in CD-patients (10μM, 100μM; 1000μM, 2000μM,) while only 1000μM spermidine increased the CD69+ the percentage of CD69+ CD8+ T-cells. A reduction of CD69 amount was observed at 2000μM exclusively in CD-patients (Figure 12: 12E, 12F for CD4+CD25; Figure 12K, 12L for CD4+CD69+; Supplementary Figure 2G–2J).

Spermidine treated CD8+ T-cell showed an enhanced percentage of autophagy and amount of LC3 per cell in 1000μM and 2000μM treated samples by CD-patients and controls. Upregulation of autophagy percentage and amount per cell was detected in the CD-patients group at a concentration of 100μM spermidine, while similar upregulation was seen in the control group already with 10μM spermidine (Figure 11G–11L, Supplementary Figure 2K, 2L).

Summary: Autophagy, percentage of CD25 and CD69 expressing CD4+ or CD8+ T-cells as well as the amount of CD25 per cell increased in a dose-dependent manner by incubation with spermidine. Only a reduction of the CD69 amount was detected for CD-patients and controls. Most of the effects were similar but some more pronounced in CD-patients than in controls.

## DISCUSSION

The main results of our present study are: i) the polyamines spermine and spermidine increase T-cell activation and autophagy, effects increased in a dose-dependent manner; ii) Spermine downregulates Th1 and Th2 cytokines in a dose-dependent manner while spermidine induces cytokine up- or downregulation depending on the dosages; iii) the effects were similar in PBMC from patients compared to controls, while a higher sensitivity regarding polyamine immune modulation in patients with cognitive decline (CD-patients) could be observed for several of the investigated parameters in comparison to controls.

As the world’s population grows older, age-related neurodegenerative diseases such as Alzheimer's Disease (AD) will increase. Despite a high number of promising targets, translation of new drugs into clinically relevant therapies failed so far [27, 28]. Thus, the interest in non-pharmacological approaches has grown, including nutritional substances like spermidine [7, 8]. So far, promising effects of polyamine substitution have been observed [7, 8], but these effect sizes are still moderate. Thus, a more thorough understanding of the mechanisms of action might enable scientists to develop targeted and more powerful substances.

While T-cells are known as important mediators in AD and their function in the pathophysiology has been investigated for more than a decade, effects of polyamines in regard to the modulation of T-cell function in cognitive diseases were not investigated. One possible mode of action of polyamines is the modulation of immune cell function, especially T-cells. In our study we found proof for this concept since spermine’s and spermidine’s effects on autophagy, cell activation and cytokine production could be observed at already very low concentrations.

### Effects on cytokine release and T-cell activation

In the present study, spermine downregulated all cytokines in a dose-dependent manner. In contrast, spermidine led to an upregulation of IL-2, IL-5, IL-9, IL-17A, INF-γ, and TNF-α for lower dosages, while high dosages upregulated IL-17A only. Therefore, our data illustrate a dose-dependent influence on immune modulation as well as differences in spermine and spermidine effects. These data are in line with Soda, Kano et al. (2005), who reported a time- and concentration-dependent influence of spermine on PBMC, regarding cytokine production [19]. Our finding that spermine and spermidine have in part opposing effects on cytokine production is confirmed by Flescher et al. who concluded that preincubation with spermidine, but not spermine, can inhibit T-cell IL-2 production upon *phytohemagglutinin* stimulation [29]. However, in contrast to Flescher et al, we found that anti-CD/anti-CD28 stimulation in combination with spermine but not spermidine inhibited IL-2 production.

Zhang and colleagues likewise demonstrated that cytokine production can affect cognitive performance: Administration of the alkaloid of the Japanese cord tree reduced the number of Th17-cells and their cytokine products, increased the number of regulatory T-cells in the AD rat model, and improved cognitive performance [30]. In our *ex vivo* experiments, both spermine and spermidine led to an enhanced T-cell activation by upregulation of early and late activation markers (CD69, CD25). Soda et al. showed that blood spermine levels inversely correlated with the surface CD11a expression upon activation on lymphocytes but not monocytes from healthy subjects. These data are in contrast to our findings of activation marker upregulation [31]. Although similar concentrations of polyamines and also PBMC were used by Soda in a cohort aged 20-70 years [31], our work focused on T-cell activation in aged cohorts (age: 74 ± 7.5 years) affected by cognitive decline compared to aged healthy controls (age: 74.3 ± 6.4 years). Young, growing cells have increased polyamine synthesis and higher intracellular polyamine concentration; however, with aging, the ability to synthesize polyamine decreases [6, 8, 32, 33]. Thus, aged cells are in higher need of extracellular supplementation. Therefore immune senescence might explain differences of our T-cell activation findings to Soda’s work [31].

### Autophagy

Phadwal et al. demonstrated decreased levels of autophagy in CD8+T-cells of aged individuals [34]. In our study, we were able to demonstrate enhanced autophagy in T-cells with higher spermidine and spermine concentrations. However, T-cells from CD-patients seemed to benefit from the polyamine treatment at lower concentration of polyamines already. While this finding is based on a low number of participants only, and therefore has to be considered in an exploratory framework, we would like to present a tentative hypothesis, to be tested in future studies: the positive effect on T-cell autophagy could not only help restore immunosenescent deficits in human patients but could also convey disease specific effects on CD-patients.

### Polyamine concentrations

The natural polyamines spermidine and spermine, are found in every living cell in high micromolar to low millimolar quantities [35]. Diet is the major source of polyamines. Taken up by the intestine they enter the circulation and cells through transport systems or endocytosis from the extracellular space [36]. In addition, polyamines can be derived from intracellular biosynthesis [36]. The dosages applied in the present study resembled the lower concentrations that are reported within blood or plasma [37–39] (at dosages 5 and 10μM), and additionally, higher concentrations (100 to 2000μM) which can be relevant within the gut. Cells, including monocyte and lymphocyte, take up polyamines from their surroundings [19]. As shown by Soda et al. intracellular concentrations of spermine in PBMCs cultured overnight with 500 μM spermine were 1.2–1.3 times higher than those cultured without the polyamines [19]. Blood concentrations after 2 months of polyamine administration increased 1.39-fold [37]. Epithelial polyamine levels in colonic epithelial cells were reported by Weiss et al. (spermidine 2.49 ± 0.26 nmol/mg; healthy controls) and correspond to 2000μM spermidine [40]. We also investigated the influence of such higher concentrations of polyamine treatment in our experiments and found major effects on the immune cells. Therefore, brain cells either have to be able to be modified by a very low polyamine upregulation or additionally, polyamine supplementation may exert its effect on brain health and function via its impact on immune cells which then interact with the brain through the gut-immune-brain axis, known to be important in cognitive diseases [41].

### Limitations

Several limitations should be considered when interpreting these findings. First, biomarker analyses of AD were not available for all patients. CSF was analyzed in 13 patients of whom 4 were biomarker positive, i. e., elevated Tau/p-Tau and decreased Ab42/40 ratio [42]. Therefore, presence of AD cannot be ascertained in all patients. In future studies, systematic assessment of samples from biomarker –positive versus biomarker-negative patients will determine if responses are modulated by biomarker status. Second, we investigated effects only *ex vivo* within a relatively small cohort of patients, limiting the statistical validity of our data with regard to subgroup analyses such as SCD, MCI and mild dementia. However, results were seen over the continuum of CD-patients. Future studies should now try to validate these findings in larger cohorts. Moreover, such studies would validate or refute the interesting finding that CD-patients seemed to benefit from polyamine treatment at lower concentration of polyamines already. Finally, effects of polyamines on proliferation were only determined indirectly, but the results gave no indication for a systematic bias within our results.

## CONCLUSIONS

To the best of our knowledge, this is the first study to provide insight into the influence of spermine and spermidine on T-cell function and their release of pro- and anti-inflammatory mediators in samples derived from CD-patients compared to samples derived from healthy aged-matched controls. Our results help to further elucidate the mechanisms underlying polyamine supplementation, demonstrating a dose-dependent polyamine—induced enhancement of T-cell autophagy activation and modulation of cytokine release.

We also found higher sensitivity against polyamine modulation of immune responses in SCD/MCI/mild dementia derived T-cells, to be validated in larger cohorts, and investigated in more depth in animal models to identify the influence of polyamines on the gut-immune-brain axis in vivo.

A more thorough understanding of the mechanism of action of polyamine supplementation on cognition may help to further improve on spermidine supplementation as a therapeutic device, possibly even in later clinical stages of AD.

## MATERIALS AND METHODS

### Patients and controls

A total of 22 patients (age: 74 ± 7.5 years) with SCD (n = 1 (5%)), MCI (n = 8 (36%)), and early-stage dementia (n =13 (59%)) were recruited at the University Medicine Greifswald. Inclusion criteria comprised age >18 years, and presence of SCD, MCI and dementia according to established criteria [43, 44]. Exclusion criteria comprised other neurodegenerative disorders, epilepsy, previous stroke, clinical sign of infection (CRP < 10mg/L), autoimmune disease, immunosuppressive drugs, malignancy or untreated medical conditions.

The cognitive tests were carried out during routine visits to the Memory Clinic of the Department of Neurology.

Healthy controls (n = 12; age: 74.3 ± 6.4 years), matched in sex and age, were recruited with identical inclusion (except cognitive status) and exclusion criteria at the Ophthalmology Outpatient Clinic at the University Medicine Greifswald. A Six-Item-Screener objectified the cognition assessment of controls. Inclusion criteria comprised a score of 5 or greater (full points: 6) [45].

Patients and controls characteristics are listed in Table 1.

**Table 1:** Patient characteristics.

**Variable**	**Patient Group (N=22)**	**Control Group (N=12)**
Age [years. mean ± SD]	74 ± 7.5	74.3 ± 6.4
Sex [as % female]	40 %	50%
**Co-morbidities**		
Depression [n (%)]	0	0
**Dementia Characteristics**		
SCD	1 (5%)	NA
MCI*	8 (36%)	NA
Dementia	13 (59%)	NA
MMSE (n=15) [xx/30. mean ± SD]	24.3 ± 4.5	NA
DemTect (n=3) [xx/18. mean ± SD]	8.6 ± 2.3	NA
MoCA (n=1) [xx/30]	23	NA
Six Item Screener [xx/6. mean ± SD]	NA	5.75 ± 0.45
anti-dementia medication [n (%)]	6 (27.0)	NA
**Social life**		
Education [Academic (%)]	9 (40.9)	NA
Duration of reported cognitive deficits [month. Mean ± SD.]	25.4 ±14.7	NA

NA: Not applicable; *3 patients had been given MCI diagnosis previously, and were not retested for this study; MMSE: Mini Mental State Examination; MoCA: Montreal-Cognitive Assessment Test.

### Blood sampling

Venous blood samples were obtained from patients and controls. Differential blood cell counts (XN9000, Sysmex, Norderstedt, Germany) and C-reactive protein (Dimension Vista. Siemens Healthcare Diagnostics, Eschborn, Germany) were determined in the central laboratory facility of the University Medicine Greifswald.

### PBMC isolation

Cell isolation of PBMC started within 1 hour of blood collection. PBMC were isolated from 18ml whole blood and anticoagulated with ethylenediaminetetraacetic acid (EDTA). The isolation was performed by density gradient using Biocoll Separating Solution (Biocoll GmbH). After isolation, PBMC were given to cell culture. Flat bottom cell culture plates (Costar® 96 Well Prod. Nr. 3596, Corning Incorporated) were coated with anti-CD3 and anti-CD28 antibodies 24 hours prior to use (Purified NA/LE Mouse anti-CD3 Clone: HIT3a / Purified NA/LE Mouse anti-CD28 Clone: CD28.2; BD Pharmingen™). Uncoated wells served as control. 200,000 PBMC were seeded in 200μL of RPMI complete medium (500ml RPMI – Sigma- Aldrich Chemie GmbH. 10ml L-Glutamine 200mM – Biochrom GmbH. 10ml Penicillin/ Streptavidin – Gibco by Life Technologies. 50ml Human AB-Serum – University Medicine Greifswald, 5ml HEPES – Gibco by Life Technologies) for 24 hours and 48 hours at 37°C and 5% CO2 supplemented with either spermine (Prod. Nr.: S4264 – Sigma Aldrich) or spermidine (Prod. Nr.: S0266 – Sigma Aldrich). Both were initially dissolved in sterile Aqua destillata (Deltamedica GmbH); 2μL of the different stock solutions were given into the cell culture per well to reach a final concentration of 5μM, 10μM, 100μM, 1000μM or 2000μM. Aqua destillata alone served as control. Since the presence of bovine serum amine oxidase [46] can result in toxicity in combination with polyamines no such supplements were used for cell culture.

### Analysis of cell activation and autophagy

Cell activation and autophagy were analyzed after 24 hours of incubation using Flow-cytometry (BD LSR-II – BD Biosciences). Cell culture wells were harvested and wells washed twice with DPBS (PAN™ Biotech) to take out all cells. Zombie NIR™ Fixable Viability Kit (BioLegend®) was used to distinguish live/dead cells. After treatment with antibodies against Fc-receptor (FcR Blocking Reagent human – Miltenyi Biotec), cells were stained extracellularly. The panel for surface markers included: anti-CD3 – Brilliant Violet 570™ - Clone: UCHT1, anti-CD8 – PE – Clone: SK1, anti-CD69 – APC – Clone: FN50 (all BioLegend®), anti-CD4 – V500 – Clone: RPA-T4, anti-CD25 – PE-Cy™7 – Clone: M-A251 (all BD Biosciences). In 4 experiments, anti-CD3- PerCP – Clone: OKT3 (BioLegend®) was used instead. Controls verified that the change of antibody did not induce alterations into the experiment. The percentages and the amounts of activation marker CD25 and CD69, determined by the median fluorescence intensity (MFI), were analyzed for CD4+ and CD8+ T-cells. Intracellular LC3 amounts stained by the FlowCellect™ Autophagy LC3-antibody based assay kit (Merck KG) served as quantification for autophagy. Permeabilization and intracellular staining was performed after surface staining following the instructions of the manufacturer.

All flow cytometry results were evaluated using FlowJo Software 10.3 (Tree Star Inc.). The gating strategy is showcased in Supplementary Figure 3A, 3B. Fluorescence minus one controls (FMOs) determined negative staining and were applied to gate the activation and autophagy markers CD25, CD69 and LC3.

### Analysis of cytokine production

After 48 hours of incubation, 100μL of supernatant were taken from each well and frozen at -80°C until analysis. The following cytokines were determined by LEGEND plex™Multi-Analyte Flow Assay Kit (BioLegend®) according to the manufacturer’s instructions: IL-2, IL-4, IL-5, IL-6, IL-9, IL-10, IL-13, IL-17A, IL-17F, IL-21, IL-22, INF-y, and TNF-α. IL-6 was always above the detection limit, while IL-21 was below – in consequence, both cytokines were excluded from analysis.

### Cell death and proliferation

Effects of polyamines on cell death were excluded by Zombie NIR FACS staining within samples gained from 2 healthy controls and 2 patients. No significant differences were observed between the different concentrations of spermine/spermidine compared to the control cell culture condition (Supplementary Figure 4A, 4B). Cell proliferation was observed by microscopy before harvesting of cells in comparable amounts in all cell culture conditions. All cells were harvested. After staining and washing for FACS analysis cells were put into an defined amount of FACS buffer. Time to reach the event count of 100.000 during sampling applying the same sampling speed did not differ significantly, which argues against effects of our chosen polyamine concentrations on proliferation (Supplementary Figure 4C, 4D).

### Statistical analyses

Statistical analyses were performed by R Studio with the packages emmeans, tidyverse, lmerTest, car and bestNormalize.

A linear-mixed-model was then used for statistical analysis to account for clustering within patients/ with patient-ID as random factor. Data sets were transformed by orderNorm using the bestNormalize package to ensure normality and homoscedasticity of residuals. This was visually checked using QQ-plots and residual-vs-fitted plots.

GraphPad-PRISM 8.3 (GraphPad Software inc., San Diego, CA, USA) was used to illustrate means and confidence intervals of the transformed data. R script is available upon request.

### Ethics approval

The study protocol was approved by the ethics committee of the University Medicine Greifswald (No. BB 026/18). All patients and control individuals were aged ≥18 years, and provided written informed consent before entering the study.

## Supplementary Material

Supplementary Figures

Supplementary Table 1
