# Supplementary material for: Spermine and spermidine modulate T-cell function in older adults with and without cognitive decline ex vivo
Source: Aging (Albany NY). 2020 Jun 30;12(13):13716–39. doi: 10.18632/aging.103527 (PMC7377836; doi:10.18632/aging.103527)
Supplement: Supplementary Figures [file aging-12-103527-s002..pdf]

SUPPLEMENTARY FIGURES

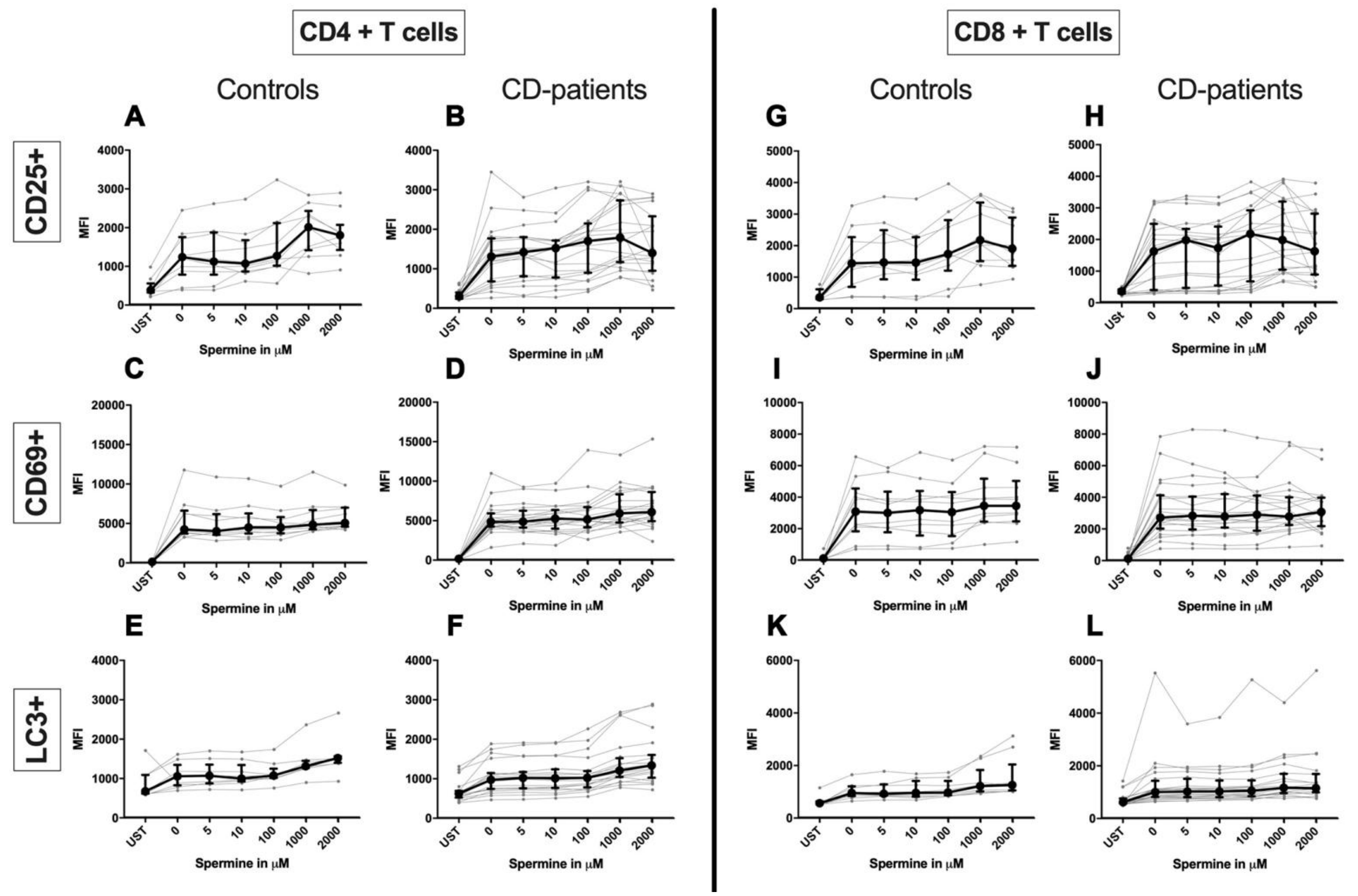

Supplementary Figure 1. Activation marker and autophagy of CD4+ T-helper-cells and CD8+cytotoxic T-cells treated with Spermine for cognitive decline patients and controls. T-cells were isolated from controls (**A**, **C**, **E**, **G**, **I**, **K**) and patients with cognitive decline (**B**, **D**, **F**, **H**, **J**, **L**). CD3/CD28 stimulated T-cells were incubated for 24 hours with 5, 10, 100, 1000, 2000 µM Spermine. The expression (measured by median fluorescence intensity; MFI) (**A**–**L**) of activation marker – CD25 (**A**, **B**, **G**, **H**); CD69 (**C**, **D**, **I**, **J**) and autophagy (LC3) (**E**, **F**, **K**, **L**). Markers were analysed on CD4+ T-cells (**A**–**F**) and CD8+ T-cells (**G**–**L**). Raw data (individual data, median + interquantile ranges) and transformed data (mean with lower and upper confidence limits) are depicted for MFI. $n_{control}$ = 12; $n_{CD\text{-}patient}$ = 20; 0.05; ** p<0.01; *** p<0.001.

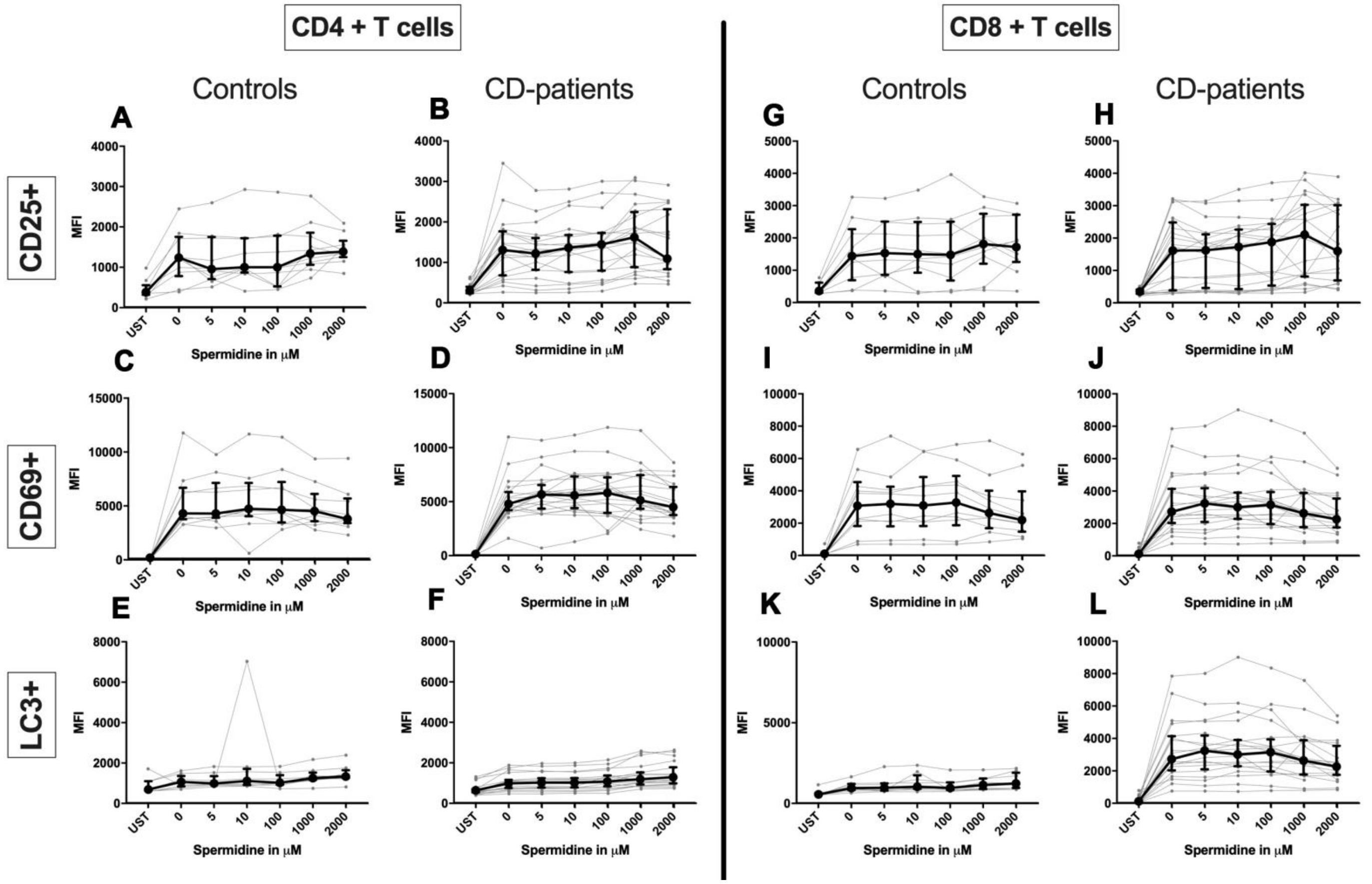

Supplementary Figure 2. Activation marker and autophagy of CD4+ T-helper-cells and CD8+cytotoxic T-cells treated with Spermidine for cognitive decline patients and controls. T-cells were isolated from controls (**A**, **C**, **E**, **G**, **I**, **K**) and patients with cognitive decline (**B**, **D**, **F**, **H**, **J**, **L**). CD3/CD28 stimulated T-cells were incubated for 24 hours with 5, 10, 100, 1000, 2000 µM Spermidine. The expression (measured by median fluorescence intensity; MFI) (**A**–**L**) of activation marker – CD25 (**A**, **B**, **G**, **H**); CD69 (**C**, **D**, **I**, **J**) and autophagy (LC3) (**E**, **F**, **K**, **L**). Markers were analysed on CD4+ T-cells (**A**–**F**) and CD8+ T-cells (**G**–**L**). Raw data (individual data, median + interquantile ranges) and transformed data (mean with lower and upper confidence limits) are depicted for MFI. $n_{control}$ = 12; $n_{CD\text{-}patient}$ = 20; 0.05; ** p<0.01; *** p<0.001.

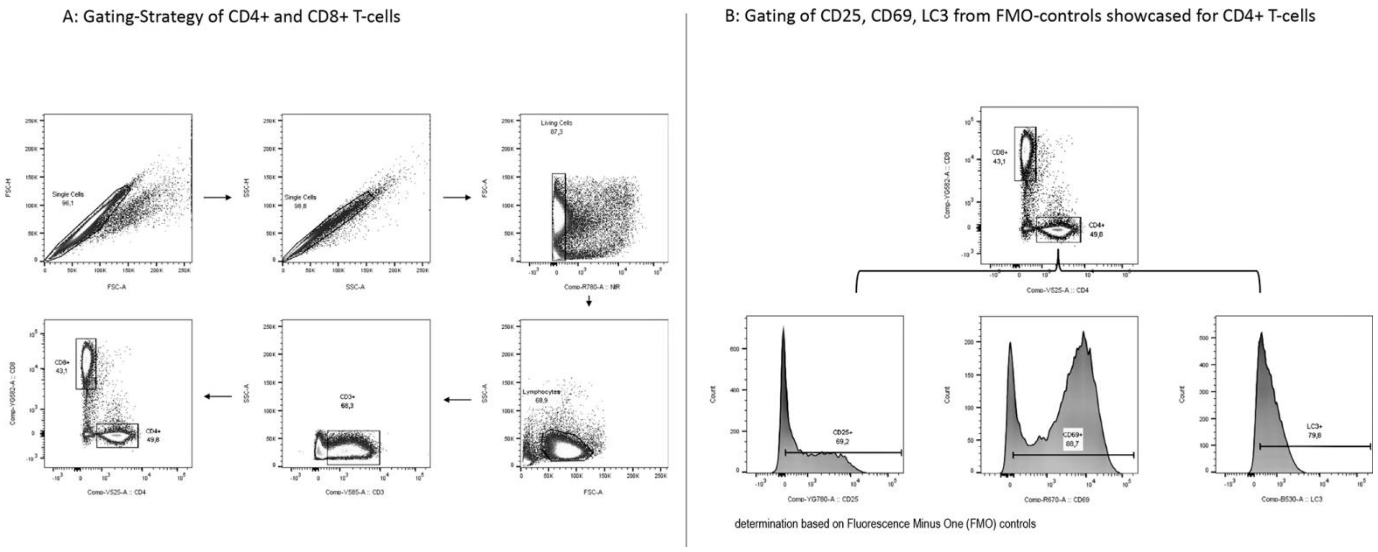

Supplementary Figure 3. Gating of CD4+ T-helper cells and CD8+ cytotoxic T-cells. Gating was performed by using FlowJo Software 10.3 (Tree Star Inc.). First single cells gating was done to eliminate duplets then death cells were excluded Zombie NIR. After gating for for CD3 positive lymphocytes, cells were distinguished into T-helper cells (CD4+) and cytotoxic T-cells (CD8+) (compare gating strategy in **A**). CD4+ T-helper cells and CD8+ cytotoxic T-cells were then analysed regarding their activation marker (CD25, CD69) and autophagy (LC3) by using fluorescence minus one controls. Gating via FMO is shown for CD4 cells in **B**.

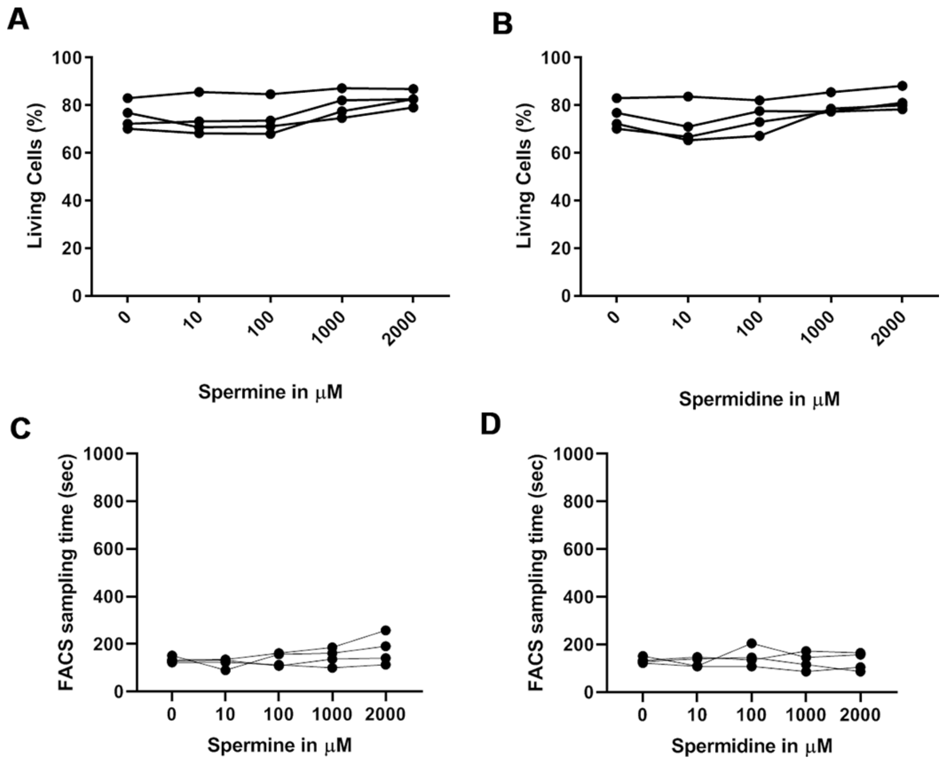

Supplementary Figure 4. Data of cell death and proliferation. We investigated cell death effects for cells derived from two patients and two control cohort subjects. Cell death was measured by Zombie NIR FACS staining for spermine and spermidine with increasing amounts of spermine/spermidine after 48h (**A**, **B**). To evaluate proliferation all cells were harvested. After staining and washing for FACS analysis cells were put into an defined amount of FACS buffer. 100.000 events where collected on the FACS machine applying the same sampling speed. We compared the time needed during sampling to reach the amount of events within the cell death control experiment. The time did not differ significantly between samples (with similar acquisition speed for all samples) (**C**, **D**).
