# Supplementary material for: Spermine and spermidine modulate T-cell function in older adults with and without cognitive decline ex vivo
Source: Aging (Albany NY). 2020 Jun 30;12(13):13716–39. doi: 10.18632/aging.103527 (PMC7377836; doi:10.18632/aging.103527)
Supplement: Supplementary Table 1 [file aging-12-103527-s001..pdf]

SUPPLEMENTARY TABLE

Supplementary Table 1.

Spermine concentration in μM	5				10				100				1000				2000			
	control		CD		control		CD		control		CD		control		CD		control		CD	
IL-2					↓	**			↓	***	↓	***	↓	***	↓	***	↓	***	↓	***
IL-4	↓	*	↓	*	↓	**	↓	*	↓	**	↓	***	↓	***	↓	***	↓	***	↓	***
IL-5									↓	**	↓	***	↓	***	↓	***	↓	***	↓	***
IL-9					↓	*			↓	***	↓	***	↓	***	↓	***	↓	***	↓	***
IL-10													↓	***	↓	***	↓	***	↓	***
IL-13			↓	*	↓	*	↓	**	↓	**	↓	***	↓	***	↓	***	↓	***	↓	***
IL-17A											↓	*			↓	**	↓	**	↓	***
IL-17F											↓	**	↓	***	↓	***	↓	***	↓	***
IL-22											↓	***	↓	***	↓	***	↓	***	↓	***
INF-y											↓	***	↓	***	↓	***	↓	***	↓	***
TNF-a			↓	**	↓	*	↓	**	↓	***	↓	***	↓	***	↓	***	↓	***	↓	***

Spermidine concentration in μM	5				10				100				1000				2000			
	control		CD		control		CD		control		CD		control		CD		control		CD	
IL-2							↑	*					↓	***	↓	***	↓	***	↓	***
IL-4													↓	*	↓	*	↓	***	↓	***
IL-5											↑	*			↓	**	↓	***	↓	***
IL-9							↑	***			↑	**			↓	*	↓	***	↓	***
IL-10													↓	*	↓	***	↓	***	↓	***
IL-13											↑	*							↓	***
IL-17A					↑	**	↑	***	↑	**	↑	***	↑	***	↑	***	↑	***	↑	***
IL-17F													↑	*					↓	***
IL-22			↓	*											↓	***	↓	***	↓	***
INF-y			↑	*	↑	*	↑	**	↑	*	↑	***					↓	*	↓	***
TNF-a			↑	**			↑	***	↑	*	↑	**	↑	*					↓	***
